# A rare case report - Urosepsis as a result of a neglected and forgotten pessary for 10 years

**DOI:** 10.1016/j.eucr.2023.102394

**Published:** 2023-04-03

**Authors:** Halit Maloku, Roena Maloku

**Affiliations:** a University of Prishtina, Faculty of Medicine, Department of Surgery, Prishtina, Kosovo, 10000, Albania; b University of Prishtina, Faculty of Medicine, Prishtina, Kosovo, 10000, Albania

**Keywords:** Pessary, Uterus, Incontinence, Urosepsis

## Abstract

A pessary is a silicone ring placed on the vagina that can improve urinary incontinence. This study reports an 83-year-old patient with a pessary discovered inside her vagina after a prior urinary incontinence treatment. The prolonged presence of the pessary in the patient's body led to a complication called urosepsis. The presence of the pessary inside the patient's vagina for an extended period was not detected by the doctor who performed the gynecological checks, nor by the patient who had forgotten about it. The pessary remained inside her for 10 years until it was discovered accidently during a hysterectomy.

## 1 Introduction

Pessaries are medical devices used to treat pelvic organ prolapse and urinary incontinence in women. They can cause many complications, such as pain, bleeding, infections, rectovaginal and vesicovaginal fistula, intestinal fistula, urosepsis, tumor, incarceration in the cervix, and even death.^1^ It's crucial to be cautious when using them, particularly in older patients who may have limited psycho-physical abilities.

This study presents a case of an 83-year-old woman who underwent a procedure for urinary incontinence and had a pessary inserted in her vagina. However, the pessary was forgotten and left inside her for ten years, which led to complications, including urosepsis, a severe infection. The doctor who performed her check-ups didn't notice it, and the patient forgot it was there. Even the radiologist who looked at her scans didn't see it. The pessary was only discovered during a hysterectomy, which was necessary due to the patient's condition.

The failure to identify the presence of the pessary resulted in the patient's severe health condition, which put her life in danger.

## 2 Case presentation

An 83-year-old woman was admitted to the hospital because she felt tired, had lower abdominal pain, trouble urinating, and a fever caused by an infection. She had been experiencing difficulty urinating for ten years, was in menopause for 25 years, had five births, and no abortions. The patient had a procedure done in her uterus, but she couldn't remember what it was.

She had been seeing a gynecologist for check-ups, but she forgot she had a pessary inserted, and her doctor didn't notice it either. For the past six months, she had also been experiencing vaginal bleeding and pus with a foul odor. During an examination at our hospital, a significant amount of pus with an unpleasant odor was observed coming from the patient's cervix, which appeared swollen.

The patient's lab results showed high white blood cell count (23,000) and high levels of C-reactive protein (CRP) (above 350), indicating inflammation in her body. The patient also had other health problems, such as diabetes and high blood pressure.

**Fig. 1:**
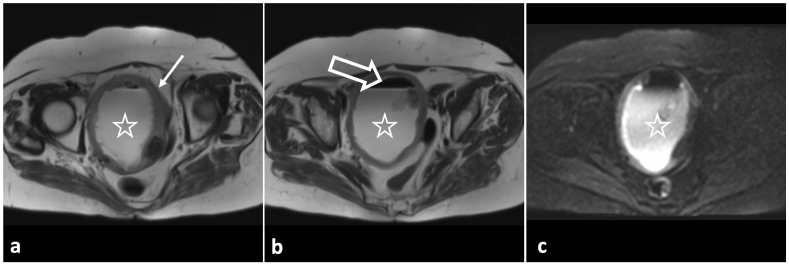
**axial MR images**. **a;** T2WI images show a thick-walled (thin arrow) cystic lesion (star). **b;** there is air-fluid leveling within the lesion (thick arrow). **c;** DWI images show diffusion restriction of the fluid component of the lesion.

**Fig. 2:**
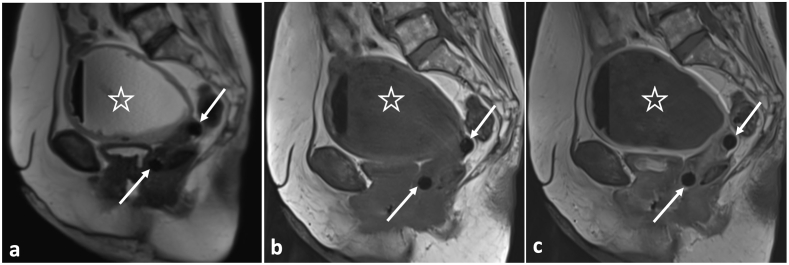
**sagittal MR images. a;** T2WI, **b;** pre-contrast T1WI. **c;** post-contrast T1WI. Thick-walled cystic lesion (star) containing air-fluid level and contrast enhancement in the wall. The ring pessary (thin arrows).

An abdominal and pelvic magnetic resonance imaging (MRI) was conducted, where it was described that the uterus had a cystic formation appearance with dimensions of 12.5 cm × 9.5 cm. The uterine cavity had a diameter of 8.5 cm and there was a presence of an air-fluid level at the level of the fundus. (Fig. 1). The MRI findings suggested a possible diagnosis of endometrial carcinoma. Although the presence of the pessary was not mentioned in the report, it is visible in the MRI images (Fig. 2).

The patient was admitted to the hospital, given antibiotics, and prepared for surgery. The severity of the patient's condition and the imaging data raised suspicion of a malignant abscessed uterus. However, a cytological examination of the fluid taken in Douglas showed no presence of cancerous cells. The decision was made to perform a total hysterectomy and bilateral adnexectomy. During the surgery, a silicone ring was found in the fornix and a large amount of pus with a foul odor was observed coming from the cervix. The patient's postoperative recovery was satisfactory, and she was released from the hospital on the fifth day after surgery.

## 3 Macroscopic description

**Fig. 3:**
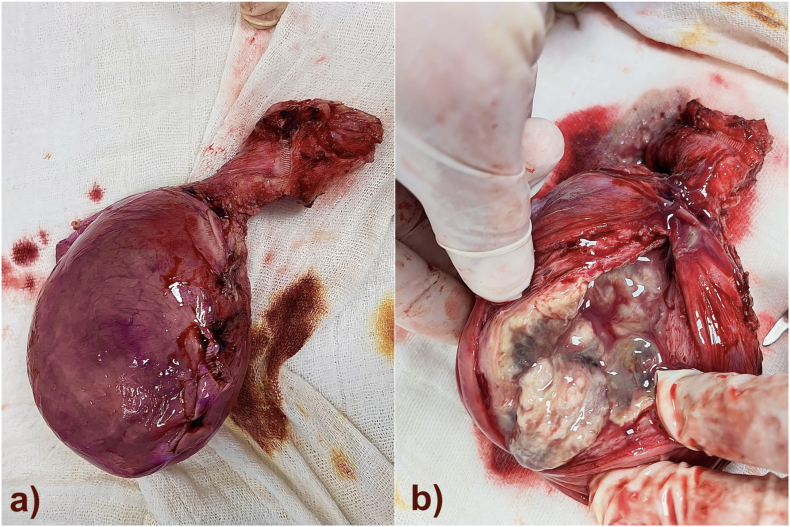
The view of the uterine cavity after the operation. a) The uterus with the cervix. b) Uterine cavity after dissection.

Uterus size: 13 × 11.5 cm, oval-shaped, with edematous, hyperemic walls, with soft consistency. In the opening there is a large mass of pus and a heavy smell with several tumor-fibrotic formations with a very strong consistency, suspicious for a malignant process (Fig. 3).

## 4 Microscopic description

Histopathological result - HP - Endometritis acuta supurativa. Adenomyosis. Leyomioma uteri. **CIN 1**.

## 5 Discussion

Carelessness can lead to rectovaginal fistula, as described in the literature, so fortunately in our case it ended only with urosepsis and successful treatment.^1^ Pessaries must be removed every 6 weeks for cleaning, whereas in our case it has not been removed or taken care of for 10 years.^2^ Nursing and professional staff should be educated on the maintenance and timely replacement of pessaries, especially for patients who are unable to do it themselves.^3^ Until 2022, only 36 cases of complications from pessaries were reported in PubMed, Embase and CINAH.^4^ In our case, there was no education on the maintenance of the pessary, leading to urosepsis. There are also cases where they are forgotten for 16 years and are asymptomatic.^5^

## 6 Conclusion

Pessaries should not be placed on people who are not psycho-physically capable of understanding the procedure and possible complications. Do not place it on patients who are unable to perform regular medical check-ups. Radiology specialists should also be attentive in cases where they come across ring-like objects in this region, for the possibility that object is the pessary with its complications. The clinician must stress proper pessary maintenance in order to avoid the serious consequences of a neglected pessary. The ideal candidate for this intervention is the patient who has a good self-care index.
